# Atomic resolution structure of EhpR: phenazine resistance in *Enterobacter agglomerans *Eh1087 follows principles of bleomycin/mitomycin C resistance in other bacteria

**DOI:** 10.1186/1472-6807-11-33

**Published:** 2011-08-17

**Authors:** Shen Yu, Allegra Vit, Sean Devenish, H Khris Mahanty, Aymelt Itzen, Roger S Goody, Wulf Blankenfeldt

**Affiliations:** 1 Department of Physical Biochemistry, Max Planck Institute of Molecular Physiology, Otto-Hahn-Straße 11, 44227 Dortmund, Germany; 2 Department of Molecular Biology, Massachusetts General Hospital, Boston, MA 02114, USA; 3 Lehrstuhl für Biochemie, Universität Bayreuth, Universitätsstraße 30, 95447 Bayreuth, Germany; 4 School of Biological Sciences, University of Canterbury, Private Bag 4800, Christchurch, New Zealand; 5 Department of Biochemistry, University of Cambridge, Cambridge CB2 1GA, UK

## Abstract

**Background:**

The phenazines are redox-active secondary metabolites that a large number of bacterial strains produce and excrete into the environment. They possess antibiotic activity owing to the fact that they can reduce molecular oxygen to toxic reactive oxygen species. In order to take advantage of this activity, phenazine producers need to protect themselves against phenazine toxicity. Whereas it is believed that phenazine-producing pseudomonads possess highly active superoxide dismutases and catalases, it has recently been found that the plant-colonizing bacterium *Enterobacter agglomerans *expresses a small gene *ehpR *to render itself resistant towards *D*-alanyl-griseoluteic acid, the phenazine antibiotic produced by this strain.

**Results:**

To understand the resistance mechanism installed by EhpR we have determined its crystal structure in the apo form at 2.15 Å resolution and in complex with griseoluteic acid at 1.01 Å, respectively. While EhpR shares a common fold with glyoxalase-I/bleomycin resistance proteins, the ligand binding site does not contain residues that some related proteins employ to chemically alter their substrates. Binding of the antibiotic is mediated by π-stacking interactions of the aromatic moiety with the side chains of aromatic amino acids and by a few polar interactions. The dissociation constant K_D _between EhpR and griseoluteic acid was quantified as 244 ± 45 μM by microscale thermophoresis measurements.

**Conclusions:**

The data accumulated here suggest that EhpR confers resistance by binding *D*-alanyl-griseoluteic acid and acting as a chaperone involved in exporting the antibiotic rather than by altering it chemically. It is tempting to speculate that EhpR acts in concert with EhpJ, a transport protein of the major facilitator superfamily that is also encoded in the phenazine biosynthesis operon of *E. agglomerans*. The low affinity of EhpR for griseoluteic acid may be required for its physiological function.

## Background

Newly emerging resistance against antibiotics is an increasing problem in the treatment of infectious disease. The situation is currently worsening at such an alarming speed that the World Health Organization decided to bring it to the spotlight by making it the topic of World Health Day in 2011 [1]. In order to overcome resistance, create opportunities for the development of novel antibiotics or enable the continued use of existing compounds, it is important to understand resistance mechanisms at the molecular level. These mechanisms are highly versatile, from simple mutation of the antibiotic's target to development of mechanisms to reduce uptake by the infectious organism or the installation of factors that destroy or in other ways deactivate the antibiotic [2]. The latter is usually achieved by horizontal gene transfer, e.g. through transmission of plasmids or transposons that carry resistance genes from one strain to the next.

On the other hand, a large number of antibiotics are produced by microorganisms themselves, which secrete these into the environment to compete with other species that colonize the same habitat. In the case of compounds with nonspecific toxicity, such as those that give rise to reactive oxygen species (ROS), the producing strain is faced with the problem of having to avoid self-poisoning. This cannot be resolved by simply destroying the antibiotic as this would contradict the purpose of synthesizing these toxins in the first place and would lead to a waste of metabolic energy instead.

One example of antibiotics with nonspecific toxicity are the phenazines, a class of bacteria-produced antibiotics that has gained increasing attention in recent years [3]. They comprise a group of over 100 compounds isolated from natural sources and several thousand derivatives that have been synthesized by chemical methods [4,5]. In addition to being able to intercalate DNA and inhibit topoisomerases, phenazines act through their redox activity, which enables them to exchange electrons with e.g. NADH, Fe^2+^/Fe^3+ ^or molecular oxygen. Whereas the reoxidation of NADH may play an important role in the survival of phenazine producers in anoxic environments, like those found in the deeper layers of biofilms, the reduction of ferric iron or O_2 _directly or indirectly leads to the generation of toxic ROS. This explains the broad specificity antibiotic activity of phenazines and also their function as virulence factors in infectious disease. For example, the blue phenazine derivative pyocyanin (5-N-methyl-1-hydroxyphenazium betaine) induces tissue damage in patients infected with a well-studied Gram-negative phenazine producer *Pseudomonas aeruginosa *[6,7] and it has been demonstrated that the immune system can clear *P. aeruginosa *infections significantly more easily if phenazine biosynthesis is impaired [8].

**Figure 1:**
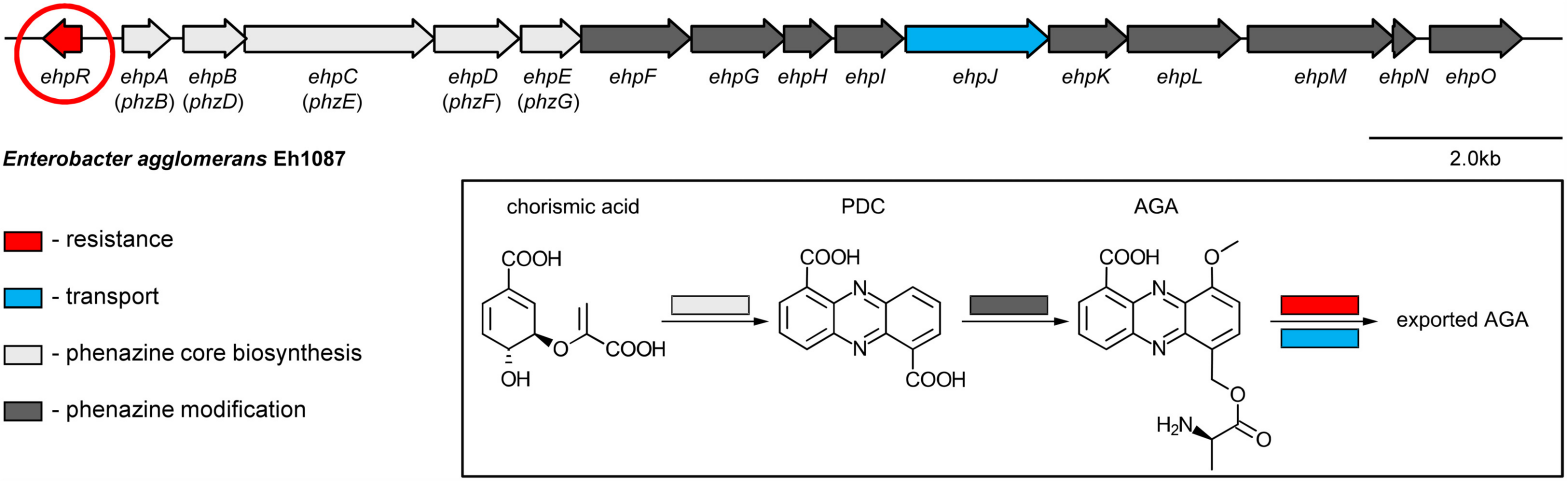
**Phenazine biosynthesis in *Enterobacter agglomerans *Eh1087**. The phenazine biosynthesis operon of *E. agglomerans *Eh1087 contains 16 open reading frames, which are required to convert chorismic acid to phenazine-1,6-dicarboxylic acid (PDC; light grey) and further to *D*-alanyl griseoluteic acid (AGA; dark grey). EhpR (red) mediates resistance to self-produced AGA. It likely acts as a shuttle that delivers AGA to the major facilitator membrane transporter EhpJ (blue).

It has been shown that *P. aeruginosa *increases the production of iron- and manganese-dependent superoxide dismutases as well as catalase in response to pyocyanin, hence protecting itself from phenazines by deactivating ROS [9]. A different mechanism of phenazine self-resistance has recently been discovered in the plant-colonizing bacterium *Enterobacter agglomerans *(previously termed *Pantoea agglomerans *and *Erwinia herbicola*) strain Eh1087. This strain is capable of controlling fireblight, a plant disease caused by the phytopathogen *Erwinia amylovora *[10], by producing the phenazine derivative *D*-alanylgriseoluteic acid (AGA) from genes carried on a 200 kB plasmid. AGA is also active against a number of other bacteria, including clinically relevant species such as *Staphylococcus aureus *[11]. It has also been isolated from a marine *Vibrio *species (SANK 73794) [12] and most likely is produced from griseoluteic acid (GA; 6-(hydroxymethyl)-9-methoxyphenazine-1-carboxylic acid), a compound that has also been found in *Pelagiobacter variabilis *[13] and more recently in an Indonesian *Streptomyces *sp. (ICBB8198) [14]. The genetic material required for AGA biosynthesis is assembled in an operon that contains the 15 open reading frames *ehpA-O *and is in part highly similar to the conserved *phz*-operon found in *Pseudomonas *and other phenazine producing species. Of the encoded enzymes, EhpA-E utilize chorismic acid to produce phenazine-1,6-dicarboxylic acid (PDC) [4], which is then converted to AGA by the remaining enzymes EhpF-O [10,15] with the exception of EhpJ, which encodes a membrane transporter of the major facilitator superfamily presumably involved in exporting AGA from the cell. In addition to the genes involved in AGA biosynthesis, the promoter of the *ehp*-operon also triggers the transcription of an additional gene *ehpR *from the second DNA strand (Figure 1). This gene encodes a protein of 129 amino acids and has been shown to give rise to resistance against AGA but not some other common phenazines like phenazine-1-carboxylic acid. When first reported in 2002, no sequence homology to other proteins could be detected [10]. In order to investigate the molecular mechanisms behind EhpR-mediated phenazine resistance, we have therefore characterized the protein and its interaction with the *E. agglomerans*-produced phenazine derivative griseoluteic acid. Our data suggest that EhpR acts as a binding protein that escorts AGA to a membrane transporter for subsequent secretion.

## Results

### EhpR belongs to the glyoxalase I/bleomycin resistance protein family

Analysis of the EhpR sequence with the fold recognition engine PHYRE [16] unequivocally assigns EhpR as belonging to the glyoxalase I/bleomycin resistance protein family. These proteins form a very large group whose members act as modifying enzymes or as binders that render toxic compounds harmless, sometimes in a metal-dependent manner. A recent sequence similarity search in April 2011 with EhpR against the Protein Data Bank [17], on the other hand, returns only one structure with an E-value below 0.5 (PDB entry 2KJZ; Lemak et al., Northeast Structural Genomics Consortium, unpublished; uncharacterized protein ATC0852 from *Agrobacterium tumefaciens*, 29% sequence identity to EhpR) despite the fact that several dozens of proteins from this family have been investigated by structural methods. Closer inspection of these structures reveals, however, that EhpR does not possess the catalytic motifs or binding residues that many of the structurally related and functionally characterized proteins utilize to interact with their substrates. This indicates that EhpR belongs to a distant and unexplored branch of this otherwise well-studied protein family.

### Structure of EhpR

EhpR behaved as a homodimer during size exclusion chromatography and crystallized in space group P2_1_2_1_2_1 _with two and one dimers per asymmetric unit in the apo and GA-cocrystallized form, respectively. The structure was solved from single-wavelength anomalous diffraction data collected at the K-edge of seleno-*L*-methionine labeled protein crystallized in the apo form. These crystals diffracted to 2.15 Å and the structure was refined to a crystallographic R-factor of 20.5% with R_free _= 25.9%. Cocrystallization with GA gave rise to highly improved crystals under the same conditions. Data were collected to 1.01 Å and the final model has an R-factor or 12.6% with R_free _= 14.6% (Table 1).

**Table 1:** Data collection and refinement statistics

	Se-SAD^2^	EhpR apo	Ehpr/GA complex
**Data collection^1^**			
Space group	P2_1_2_1_2_1_	P2_1_2_1_2_1_	P2_1_2_1_2_1_
Cell dimensions			
*a, b, c *(Å) α, β, γ (°)	71.6, 78.9, 87.2 90, 90, 90	72.6, 79.3, 87.9 90, 90, 90	36.5, 79.8, 82.9 90, 90, 90
Wavelength	0.979147	0.934	0.934
f' (electrons)	-8.2	-	-
f'' (electrons)	5.1	-	-
Resolution (Å)^3^	20 - 2.7 (2.8 - 2.7)	20 - 2.15 (2.25 - 2.15)	20 - 1.01 (1.11 - 1.01)
*R*_sym_(I) (%)^4^	13.7 (52.8)	5.0 (35.9)	4.0 (35.9)
*R*_merge_(F) (%)^5^	7.3 (33.4)	5.6 (29.4)	6.4 (36.6)
< I/σ(I) >	15.3 (4.2)	23.0 (5.1)	19.0 (4.0)
Completeness (%)	99.7 (100)	98.1 (92.5)	98.5 (95.5)
Redundancy	7.7 (7.6)	6.8 (5.0)	4.3 (3.1)
**Refinement**			
Resolution (Å)		20 - 2.15 (2.20 - 2.15)	20 - 1.01 (1.02 - 1.01)
No. reflections		26266 (1800)	125498 (3663)
*R*_work _(%)		20.5 (29.2)	12.6 (23.7)
*R*_free _(%)		25.9 (30.1)	14.6 (24.3)
No. atoms/*B*-factors (Å^2^)^6^			
Protein		3928/73	2359/13
Ligands/ions		-	21/17
Water		66/44	499/29
R.m.s deviations			
Bond lengths (Å)		0.020	0.022
Bond angles (°)		1.733	2.085
			
**PDB entry code**		3SK1	3SK2

^1^All data sets were collected from single crystals.

^2^Data collections statistics for MAD data refer to unmerged Friedel pairs.

^3^Values in parentheses refer to the highest resolution shell.

^4^R_sym_(I) = (Σ_hkl _Σ_i _|I(h)_j _- <I(h)>|)/(Σ_hkl _Σ_i _I(h)_j_), where I(h)_j _is the measured diffraction intensity, <I(h)> is its average and the summation includes all observations.

^5^R_merge_(F) = (Σ_hkl _SQRT (n/(n - 1)) Σ_i _|F(h)_j _- <F(h)>|)/(Σ_hkl _Σ_i _F(h)_j_) is a redundancy-independent merging R-factor of structure factor amplitudes. Symbols and indices are analogous to those in the calculation of R_sym_, n is the number of observations of reflection h and SQRT indicates the square root [43].

^6^The contribution of TLS parameters to B-factors of the EhpR apo structure has been removed with TLSANL [44].

**Figure 2:**
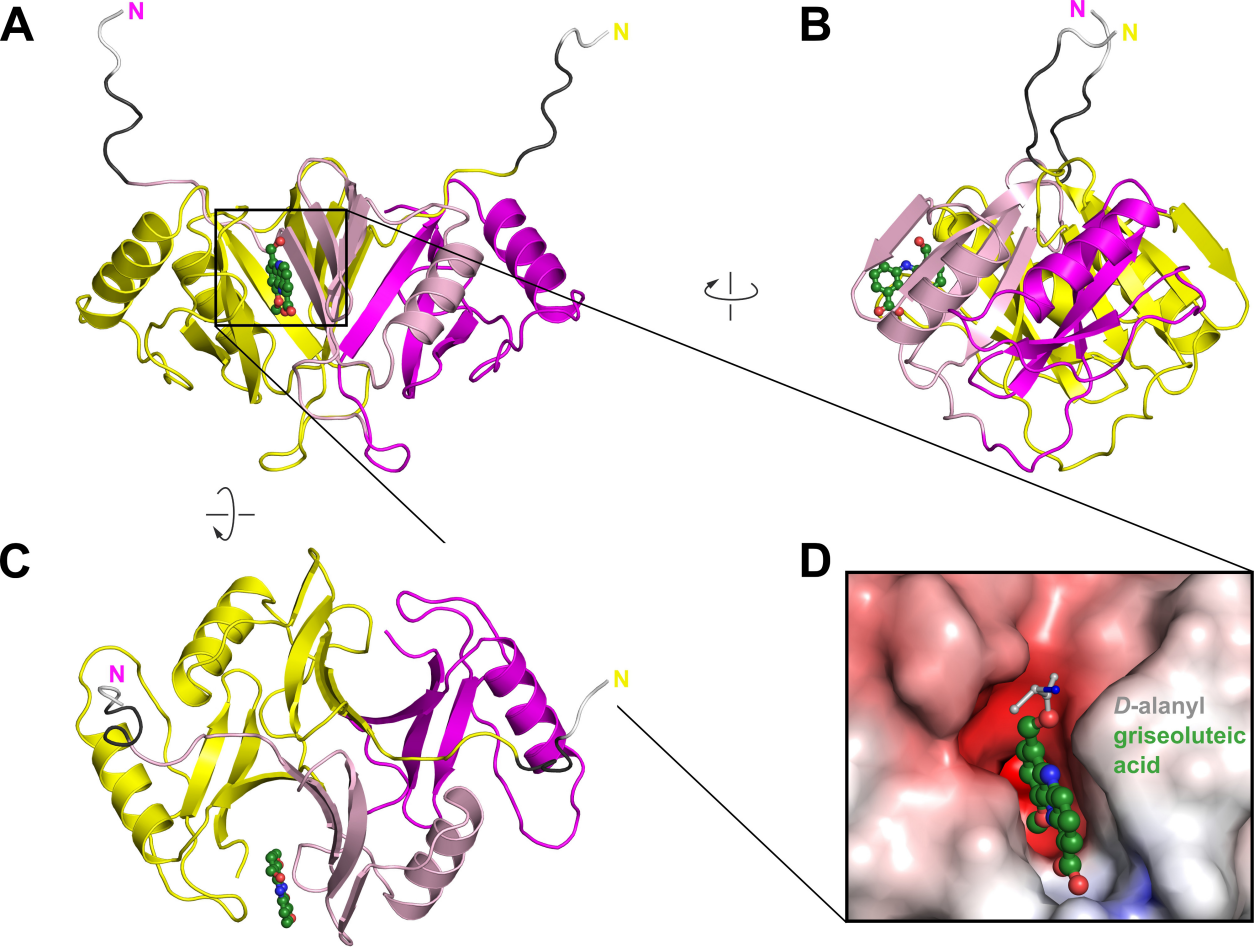
**Overall structure of EhpR**. (A-C) Three perpendicular views of the EhpR homodimer. Each monomer consists of two similar domains, shown in magenta and pink for one chain. The N-termini are arm-exchanged between the two chains and form extended antennae that are only visible in the high-resolution EhpR/griseoluteic acid complex (dark grey). Griseoluteic acid is shown in ball-and-stick representation, residues left behind after thrombin removal of the N-terminal His_6_-tag used for affinity purification are shown in white. (D) Molecular surface around the griseoluteic acid binding site, colored by electrostatic potential calculated with APBS [42]. The *D*-alanyl moiety of AGA has been modeled (grey).

The EhpR monomer consists of two βαββ'β fold units (' indicating antiparallel orientation with respect to the other strands) that are typical for members of the glyoxalase I/bleomycin resistance protein family. Because of this internal symmetry within the monomer, there are two principal ways of forming the dimer, which can be distinguished by the interaction of α-helices from two modules. These α-helices either contact each other within the monomer or at the monomer/monomer interface [18]. In EhpR, the helices interact within the monomer and this leads to the formation of two 8-stranded β-sheets that are each made up from two βαββ'β units where each monomer contributes one unit (Figure 2A-C). This generates two conspicuous half-β-barrels that contain the ligand binding or active sites of EhpR and related proteins. Also noteworthy in the EhpR dimer are the extended N-termini, which give rise to an "arm exchange" between the two monomers. While these arms are too flexible to be traced beyond G6 in the apo structure, all residues including those remaining from the N-terminal His_6_-tag after thrombin cleavage could be traced in the atomic resolution data set. In this structure the N-termini extend far beyond the fold core and have the appearance of "antennae". It is tempting to speculate that they may be required for the function of EhpR in providing resistance towards self-produced phenazines.

A DALI search [19] for similar structures in the Protein Data Bank PDB [17] retrieves more than 60 different proteins, many of which have not been characterized functionally. The structure-derived sequence identity of EhpR to these entries is generally below 20%, showing that EhpR stems from another part of the family tree that has not been investigated to date. The most similar structure is the fosfomycin resistance protein FosA from transposon *Tn2921 *(PDB entry 1NPB) [20] with an rmsd of 2.2 Å for 116 residues and a sequence identity of 14%. FosA is a metal-dependent hydrolase, and the residues required for its activity are not conserved in EhpR.

### Binding of griseoluteic acid

**Figure 3:**
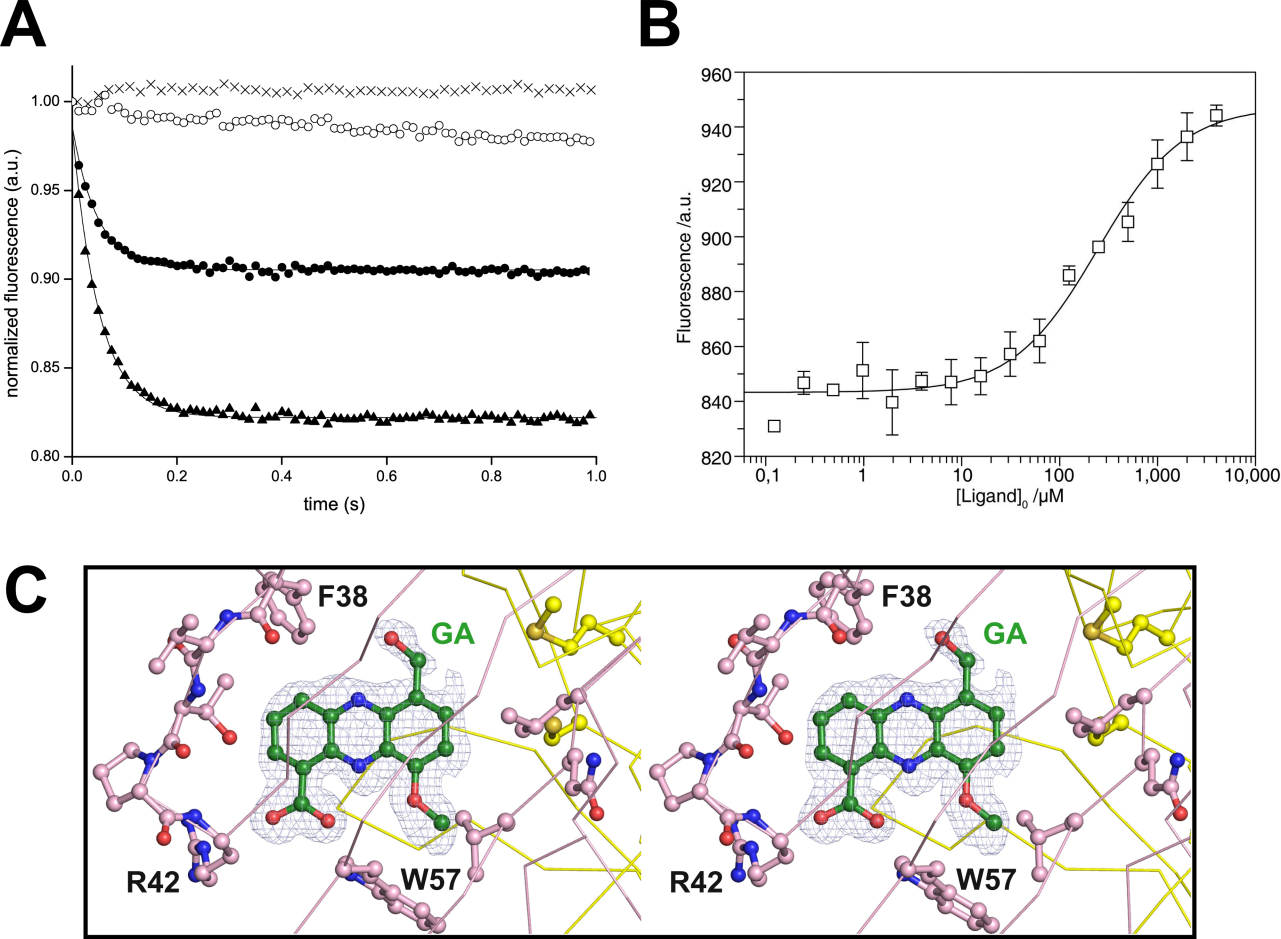
**Griseoluteic acid binds EhpR**. (A) Stopped-flow transient kinetic measurements demonstrate binding of griseoluteic acid to EhpR. Incubation of EhpR (5 μM) with an excess of griseoluteic acid (black circle: 250 μM; black triangle: 500 μM) leads to a time-dependent decrease of tryptophan fluorescence, whereas no change is observed in the absence of griseoluteic acid (cross) or EhpR (empty circle). (B) Microscale thermophoresis measurements of 25 nM fluorescein-labeled His_6_-EhpR incubated with the indicated amounts of griseoluteic acid. The relative fluorescence in the thermophoresis phase of the experiment has been plotted against the concentration of the ligand. (C) Stereo plot of |F_O_-F_C_| difference electron density at the ligand binding site of the high-resolution EhpR/griseoluteic acid (GA) complex before incorporation of the ligand, displayed at 3.5 σ.

Interaction studies were performed with griseoluteic acid (GA) since *D*-alanylgriseoluteic acid was not available in sufficient quantities for this study. While it was not possible to detect binding of GA by isothermal titration calorimetry, a time-dependent decrease of protein tryptophan fluorescence in a stopped-flow experiment indicated interaction between GA and EhpR. However, the signal could not be saturated with a large excess of GA (250 or 500 μM GA vs. 5 μM EhpR), indicating that the interaction is relatively weak (Figure 3A). This was confirmed in microscale thermophoresis measurements, where the dissociation constant K_D _between fluorescein-labeled EhpR and GA was quantified as 244 ± 45 μM (Figure 3B).

Preincubation with GA nevertheless led to highly improved crystals and additional electron density reveals the presence of GA in one of the ligand binding sites (Figure 3C). In contrast to the apo structure, which crystallized with two EhpR dimers in the asymmetric unit, the asymmetric unit of GA-cocrystallized EhpR contains only one dimer despite having been obtained with the same precipitant. The occupied ligand binding site resides in an area of crystal contacts, but neighboring molecules do not directly contribute to GA binding. The very high resolution of the ligand complex reveals details that are not discernable in the apo structure, e.g. alternative orientations of several backbone carbonyl groups and the presence of two alternative traces in Y105 - T106 (not shown).

**Figure 4:**
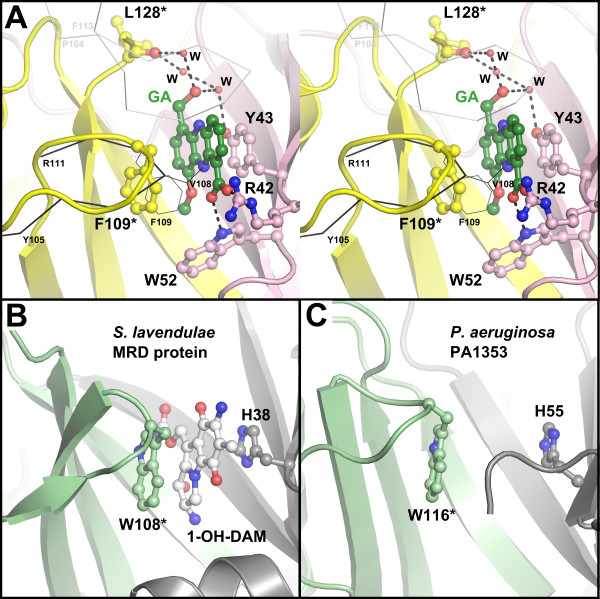
**Interactions between griseoluteic acid and EhpR**. (A) Stereo figure of the ligand binding site of EhpR with bound griseoluteic acid (GA). An asterisk indicates residues from the second monomer. Amino acids that block binding in unoccupied binding sites of the apo and complex structure (V108, F109) have been superimposed and are shown in thin black lines. Residues from a crystallographic neighboring molecule are shown in thin white lines. (B) Ligand binding site of mitomycin C resistance protein MRD from *Streptomyces lavendulae *in complex with 1,2-*cis*-1-hydroxy-2,7-diaminomitosene (1-OH-DAM; PDB entry 1KLL[21]). This related protein binds its ligand through a similar π-stacking as EhpR. (C) Aromatic side chains in the potential ligand binding site of the uncharacterized *Pseudomonas aeruginosa *protein PA1353 (PDB entry 1U6L).

In common with other proteins of this enzyme family the ligand binding sites of EhpR are located in the half-barrels that form from β-strands of both monomers. In EhpR, the binding site adopts the shape of a cleft (Figure 2D), and the interactions with GA involve hydrogen bonds between GA's carboxylate group and the side chains of R42 and W57 together with water-mediated contacts of the hydroxyl group with the side chain of Y43 and the carbonyl of L128* (* indicating residues of the second monomer, Figure 4A). A large contribution to complex formation seemingly results from a π-stacking interaction of the phenazine ring system with the side chain of Y43. Similar to EhpR, binding through π-stacking is also found in the related mitomycin C binding protein from *Streptomyces lavendulae *(PDB entry 1KLL), where two aromatic side chains hold the ligand in a clamp-like fashion [21] (Figure 4B). Indeed, in EhpR the phenyl ring of F109* is located on the opposite face of the phenazine moiety, yet the position and orientation is not optimal for π-stacking with the ligand. While the apo and complex structure are otherwise highly similar (average rmsd < 0.6 Å over the complete monomer), F109* undergoes a significant conformational and positional change on ligand binding. It adopts a different rotamer and moves together with the loop from E103 to G110, which leads to the formation of an open conformation of the ligand binding site with respect to the apo structure. This movement is required to unblock the binding site and suggests that ligand binding follows a multi-step mechanism. These steps could, however, not be resolved in the stopped-flow experiments carried out in this study, since all time traces could satisfyingly be fitted to single exponentials (not shown).

Structural changes in the loop from E103 to G110 establish new crystal contacts, which explains the generation of a new crystal lattice in the cocrystallization experiment (Figure 4A). Other ligand-induced changes involve the C-terminal residue D129, which becomes partially disordered on ligand binding.

Only one binding site was occupied in the structure obtained by cocrystallization. The other center remains blocked by the side chains of V108 and F109, which adopt the same conformation as in the apo structure here. It is not clear at present if this non-symmetrical behavior is the consequence of anti-cooperativity between the two binding sites or results from the weak binding between EhpR and GA, which may require additional stabilization of the open conformation by the newly established crystal contacts mentioned above.

## Discussion

The investigation of resistance mechanisms against self-synthesized broad-spectrum antibiotics in microorganisms is an interesting field of study because it can provide insight into how resistance against these compounds may emerge even before they are applied in the clinic. In this study, we have analyzed the phenazine resistance protein EhpR from *Enterobacter agglomerans *Eh1087, a strain that can be employed for biological control of bacteria-induced disease in several economically important plants.

The crystal structure of EhpR demonstrates that the protein belongs to the family of glyoxalase I/bleomycin resistance proteins. Even though it possesses only low sequence homology to the better studied members of this family, the atomic resolution crystal structure of EhpR in complex with griseoluteic acid shows that the protein shares common principles with mitomycin C and bleomycin resistance protein in binding the antibiotic, namely a π-stacking sandwich interaction to hold the flat aromatic molecule (Figure 4A/B). Because the binding site does not contain residues that catalyze chemical conversion in other related proteins like glyoxalase I or fosfomycin resistance protein FosA/X, EhpR most likely acts as a chaperone involved in the secretion of phenazine antibiotics produced by *P. agglomerans*. It is tempting to speculate that the cognate transporter of EhpR is EhpJ, which is a major facilitator superfamily transport protein also encoded within the phenazine biosynthesis operon of this strain (Figure 1) [10].

It is interesting that the binding of GA is relatively weak. While we presently cannot exclude that AGA binds more tightly, a model of this complex based on the structure with GA argues against this because the *D*-alanyl group projects to the surface of EhpR with no strong interactions discernable (Figure 2D). In addition, the finding that the binding site is blocked by F109 in the apo structure indicates that the ligand needs to induce structural rearrangements. This will also make binding more difficult than in the related mitomycin C and bleomycin resistance proteins, whose binding sites are not blocked in the unliganded form (compare e.g. PDB entries 1KLL and 1KMZ, [21]). In accordance with this, the reported dissociation constants for mitomycin C resistance protein and its ligands are between 6.3 and 31 μM [21], approximately one order of magnitude smaller than the K_D _between EhpR and GA measured here. Weak binding may be a desired property of EhpR, since the protein also needs to be able to release its ligands once it reaches the membrane exporter and the affinity needs to be tuned to the intracellular AGA concentration in *E. agglomerans *to ensure efficient shuttling of the antibiotic. While the intracellular concentration of AGA is not known, other phenazine producers generate high amounts of phenazines and can be optimized to produce several grams of phenazines per liter of culture (corresponding to > 10 mM concentration) [22], indicating that the low affinity for GA observed here may just be optimal for the hypothesized chaperone function of EhpR.

Finally, the interaction between GA and EhpR seems relatively nonspecific, with only a few hydrogen bonds being formed between the protein and the compound. This has also been noted for mitomycin C resistance protein MRD [21] and it will be interesting to study whether EhpR can also bind other aromatic molecules and export them from the cell. Because of this anticipated non-specificity, it is also possible that related proteins in other microorganisms can render these strains resistant to phenazines. In this respect, it is interesting to note that the genome of the well-studied phenazine producer *Pseudomonas aeruginosa *encodes over 20 proteins of this family. The structures of four of these proteins have been determined, but with the exception of fosfomycin resistance protein PA1129 (PDB entry 1NNR) [23], their functions have not been investigated experimentally (*P. aeruginosa *genes PA1353, PA1358 and PA2721 with PDB entries 1U6L, 1U7I and 1U69[24], all deposited by structural genomics centers). However, since some of these uncharacterized proteins possess the two aromatic residues required for the π-stacking sandwich binding of aromatic ligands (Figure 4C), they may be capable of binding phenazines and other related aromatic compounds. This may provide a means of resistance that works in addition to the increase in superoxide dismutase and catalase activity described previously [19]. The low specificity of these binders may also provide a basis for the rapid development of new resistance. In this regard, it is interesting to note that mitomycin C-binding proteins from *Streptomyces *spp. have been found to also bind the structurally unrelated bleomycin, which is kept in an inactive state by a related yet metal-dependent protein in bleomycin-producing streptomycetes [25]. Clearly, this aspect will have to be investigated further.

## Conclusions

*Enterobacter agglomerans *strain Eh1087 generates the phenazine antibiotic *D*-alanyl griseoluteic acid to compete with other microorganisms in its habitat. In order to protect itself against the toxic action of this compound, the bacterium produces the resistance protein EhpR together with enzymes required for phenazine biosynthesis. EhpR belongs to the glyoxalase I/bleomycin resistance protein family, whose members have a wide variety of functions extending from simple binding of toxic small molecules to their chemical conversion through enzymatic activity. The structure of EhpR in complex with griseoluteic acid suggests that it probably acts as a binder that works in tandem with a membrane-spanning exporter protein. This exporter may be EhpJ, which is also found in the phenazine biosynthesis operon of *E. agglomerans*. A relatively weak affinity for griseoluteic acid presumably reflects a high level of phenazines generated by this strain. Because the interaction between ligand and protein relies on relatively unspecific interactions, mainly consisting of π-stacking with two aromatic amino acids, EhpR may be capable of binding other aromatic compounds, and related proteins from other species may be able to bind phenazine derivatives.

## Methods

### Production of recombinant EhpR

*ehpR *(UniProtKB entry Q8GPH6) was amplified from a pBluescript plasmid with primers ehpR-for (5'-GGACCTCCATATGACTGATCTAGCTGGCCC-3') and ehpR-rev (5'-TTGGATCCTCAATCAAGCGGGCAGACC-3') and then cloned into pET15b (Merck Biosciences). Heterologous expression employed *E. coli *Rosetta pLysS [DE3] (Merck Biosciences) in LB medium at 37°C induced with 1 mM IPTG. The protein was purified on immobilized Ni^2+^, using HiTrap chelating resin, followed by cleavage of the N-terminal His_6_-tag with thrombin and final size exclusion chromatography on Superdex S75 (GE Healthcare) equilibrated with 20 mM TRIS-HCl pH 8.0, 150 mM NaCl. Fractions containing pure protein were pooled, concentrated to 20 mg/ml and stored at -80°C until further usage.

Seleno-*L*-methionine labeling was achieved by suppressing methionine biosynthesis in synthetic media supplemented with Se-L-methionine [26].

### Crystallization, data collection, structure solution and refinement

Initial crystallization conditions were determined with Crystal Screen and Crystal Screen 2 from Hampton Research. The optimized setup consisted of a hanging drop of 1 μl protein solution at 20 mg/ml EhpR mixed with 1 μl reservoir (27 - 30% PEG 4000, 0.2 M ammonium acetate, 0.1 M sodium citrate pH 5.6) equilibrated against 500 μl reservoir at room temperature. Crystals of the seleno-*L*-methionine labeled protein were obtained under similar conditions. For cocrystallization with griseoluteic acid, a suspension of the ligand at a nominal concentration of 5 mM was prepared in 100 mM TRIS-HCl pH 8.5 and then mixed 1:1 with protein solution at 40 mg/ml EhpR on ice for one hour. Griseoluteic acid was prepared as described previously [10].

Diffraction data were collected at 100 K at beamlines ID14EH2, ID14EH3 and ID29 of the European Synchrotron Radiation facility (ESRF Grenoble, France). Cryoprotection was not required. All data were indexed, integrated and scaled with the XDS package [27]. The structure of the apo form was solved from SAD data collected at the K-absorption edge of a crystal prepared from seleno-*L*-methionine labeled protein. Anomalous differences were extracted with XPREP (Bruker Analytical X-ray Solutions) and selenium atoms were located with SHELXD [28]. Phasing was achieved in SHARP [29] and the correct hand was discerned after solvent flattening with SOLOMON [30] and DM [31] from the CCP4 suite [32]. Bones were edited in O [16] and used to superimpose a similar structure that had previously been identified with PHYRE [33]. This was then used to derive a monomer mask and NCS operators using MAMA [34], LSQMAN [35] and IMP from the RAVE package [32]. After overlap removal with NCSMASK from the CCP4 suite, DM was employed for 4-fold NCS-averaging, which greatly improved the quality of the electron density map. The model was traced in O [36] and COOT [37]. REFMAC5 [38] was employed for maximum likelihood refinement, defining each single chain as a TLS body.

The high-resolution EhpR/GA complex was solved by molecular replacement with MOLREP [39], using a dimer of the apo structure as search model. Refinement followed a similar procedure as for the apo form, using ligand restraints dictionaries generated with PRODRG [40] for REFMAC5 and with eLBOW [41] for phenix.refine [38]. phenix.refine was employed for the final rounds of refinement, which included the determination of anisotropic displacement parameters. The restraints for griseoluteic acid were tightened to preserve the geometry of the ligand in the course of refinement with phenix.refine.

Figures were prepared with PyMOL [41].

Full data collection and refinement statistics are provided in Table 1.

### Stopped flow experiments

Association kinetics of EhpR with griseoluteic acid were observed at 25°C in a stopped flow apparatus (Applied Photophysics) by following changes in the tryptophan fluorescence of the protein (λ_ex _= 298 nm; λ_em _> 320 nm (cut-off filter)) for 1 second. EhpR was applied at a final concentration of 5 μM, the concentration of GA was varied between 250 and 500 μM. Both protein and ligand were dissolved in 50 mM TRIS-HCl pH 7.5, 5 mM MgCl_2_. Individual stopped-flow traces were fitted to a single exponential to obtain pseudo first-order rate constants (k_obs_). While these experiments demonstrated that EhpR and GA interact, no linear relationship between ligand concentration and k_obs _was observed. As a consequence, it was not possible to determine the affinity or the association rate constant of the reaction.

### Microscale thermophoresis measurements

His_6_-tagged EhpR at a concentration of 118 μM was labeled with fluorescein isothionate (FITC) at a protein:reagent ratio of 1:0.9 in 0.1 M Na_2_CO_3 _pH 9.3 at 298 K for one hour. Unreacted FITC was removed with a NAP5 sephadex column (GE Healthcare) primed with 0.1 M TRIS-HCl pH 8.5, resulting in a label/protein ratio of 0.8.

A series of 15 1:2 dilutions from 4 mM to 122 nM GA in 25 nM FITC-EhpR solution was prepared and thermophoresis at 298 K was measured for 30 s in a Monolith NT.115 device (NanoTemper Technologies GmbH), using 100% infrared laser power. Data of three independent runs were averaged and fitted to a hyperbolic function using Grafit (Erithacus Software).

## Authors' contributions

SY, AV, SD, HKM, AI, RSG and WB designed the research. SY, AV, SD, AI, RSG and WB performed the research. WB wrote the paper. All authors have read and approved the final manuscript.
